# Editorial: Advancing the understanding of surgical management for degenerative spine conditions

**DOI:** 10.3389/fsurg.2022.1099978

**Published:** 2023-01-09

**Authors:** Runhan Fu, Chi Zhang, Wei Wang, Wei Zeng, Hengxing Zhou, Shiqing Feng, Lingxiao Chen

**Affiliations:** ^1^ Department of Orthopaedics, Qilu Hospital of Shandong University, Shandong University Centre for Orthopaedics, Advanced Medical Research Institute, Cheeloo College of Medicine, Shandong University, Jinan, China; ^2^ School of Physics and Mechanical and Electrical Engineering, Longyan University, Longyan, China; ^3^ The Second Hospital of Shandong University, Cheeloo College of Medicine, Shandong University, Jinan, China; ^4^ Faculty of Medicine and Health, The Back Pain Research Team, Sydney Musculoskeletal Health, The Kolling Institute, University of Sydney, Sydney, Australia

**Keywords:** degenerative spine conditions, spine surgery, evidence-based, surgical techniques, prognosis

**Editorial on the Research Topic** Advancing the understanding of surgical management for degenerative spine conditions

Degenerative spine conditions are common in adults, especially among the elderly. In parallel with the aged tendency of population worldwide, the prevalence of degenerative spine diseases has been increasing. There has also been an increasing trend in spine surgery worldwide (1–3). However, current understanding of surgical treatment for degenerative spine conditions is insufficient: on the one hand, the results of some studies indicated that surgery might not be better than nonsurgical treatment for some patients (4, 5); on the other hand, advancements in surgical techniques during recent years provide surgeons diverse options to perform the operations, however, evidence is lacking for which one should be preferred. In this Research Topic, a number of valuable articles involving basic knowledge, treatment (comparison of surgical procedures and learning curve of surgical technique) and prognosis (prediction model, prognostic factors and complications after surgery) of degenerative spine disorders have been published.

Learning the basic knowledge of spinal disorders helps to give us an overall understanding of surgical treatment. Several papers in this Research Topic gave us their understandings of pathogenesis of some spine diseases. The reason why cervical sagittal curvature of certain patients will be lordotic after laminoplasty is unclear. As pointed out by *Qian* et al. that may because the laminoplasty releases dorsal spinal cord from compression in pinching cervical spondylotic myelopathy (PCSM). *Yang* et al*.* took advantage of finite element analysis of CT images and found the maximum stress in involved segments of cervical spondylotic myelopathy (CSM) was higher compared with the control group. Two papers summarized theoretical knowledge of certain spine diseases. *Xu* et al*.* gave a detailed and authoritative review for ponticulus posticus, including the epidemiology, pathology, anatomy, clinical presentation, radiographic examination and surgical significance of ponticulus posticus. *Mei* et al*.* gave us a rare case of camptocormia related to Parkinson's disease and reviewed the literature on camptocormia.

A number of papers in the current Research Topic focused on comparing different surgical procedures used in spine surgery. *Wasinpongwanich* et al*.* in their systematic review and meta-analysis, summarized fusion rate, operative time, clinical outcomes, complications (e.g., total adverse events and revision rate) for transforaminal lumbar interbody fusion (TLIF) vs. other techniques used in lumbar spine diseases. Focusing on elderly patients with single-level thoracolumbar severe osteoporotic vertebral compression fracture (sOVCF), *Zhou* et al*.* provided evidence for the effects of percutaneous kyphoplasty (PKP) with vs. without posterior pedicle screw fixation (PPSF) on long-term spinal sagittal balance. Due to limited guideline information on whether indirect decompression is sufficient after oblique lumbar interbody fusion (OLIF), *Tseng* et al*.* compared the effectiveness of the indirect decompression by OLIF with direct posterior decompression among lumbar foraminal stenosis patients. Radiofrequency denervation, as a common interventional treatment for chronic low back pain, has different emerging types such as pulsed radiofrequency denervation. In their systematic review, *Li* et al*.* comprehensively reviewed literature on radiofrequency denervation therapy in treating facet joint-derived chronic low back pain and compared efficacy of different radiofrequency denervation interventions using network meta-analysis.

Understanding the learning process of surgical techniques is beneficial to surgeons who hope to master one surgical technique. For the past few years, unilateral biportal endoscopic (UBE) has become a popular technique for spinal surgery. However, even for skilled spinal surgeons, there may be obstacles in the learning process of UBE technology. To this end, *Chen* et al*.* evaluated the learning curve of UBE using the cumulative summation (CUSUM) method analysis.

Prognosis research is of great importance in the context of current spine surgery. Several papers published in this Research Topic developed prediction model and investigated prognostic factors in different kinds of spine surgery. Clinical prediction models have plenty of applications in clinical practice. For instance, clinical prediction models help us to decide whether we need further testing, whether we need to start a treatment and which treatment need to be performed (6). *Briguglio* et al*.* tried to develop a hemoglobin-based prediction model to predict long-term recovery after spine surgery but regrettably, this model may not be reliable due to the low specificity. Nevertheless, as indicated by *Briguglio* et al*.* preoperative hemoglobin, interestingly, is one of the key laboratory biomarkers to predict long-term recovery after spine surgery. Identifying prognostic factors for patients who received spine surgery is also of great importance. By finding out factors which could provide prognostic information for patients, we may forecast the future outcomes in patients with a particular health condition and thus choose more suitable treatment under a specific situation. The risk factors for postoperative shoulder imbalance are rarely reported in adult scoliosis (AS). Hence, *Ke* et al*.* performed a detailed assessment of risk factors related to radiography in AS patients who underwent correction surgery. *Deng* et al*.* gave a comparison of sagittal balance and functional outcomes in lumbar fracture surgery patients using different intermediate pedicle screws with different insertion depth. *Wei* et al*.* examined risk factors of bone graft nonfusion for spinal tuberculosis patients who underwent lesion removal, bone graft fusion and internal fixation.

Complications after surgery also deserve more attention. Focusing on postoperative cage subsidence, a common complication after spine surgery, *Jin* et al*.* compared the subsidence rate in zero profile anchored spacer (ROI-C) and conventional cage and plate construct (CPC) in patients undergoing anterior cervical decompression and fusion (ACDF). Cases series by *Florence* et al*.* provided eight cases who had hardware complications after placement of interspinous process devices (IPDs) and gave us experience in management of high risk IPD patients.

This Research Topic also included papers related to other orthopedic surgery, which give an additional view for spine surgery. For instance, *Xu* et al*.* developed a new surgical plan for adults with tibial-eminence fracture (TEF) and assessed the clinical effectiveness of day case arthroscopic-surgery treatment. Interestingly, thromboelastography (TEG) markers could forecast the occurrence of ecchymosis after total knee arthroplasty (TKA), as found by *Chen* et al.

We sincerely thank all authors who contributed to the current Research Topic “Advancing the Understanding of Surgical Management for Degenerative Spine Conditions”. We appreciate the reviewers' valuable comments and constructive suggestions. Also, we express our gratitude to the editorial team for their support.

We unfeignedly hope that articles in this Research Topic will help surgeons make the right decisions and inspire researchers to give a further exploration of surgical management for degenerative spine conditions.
